# Supplementary material for: Factors Associated with Prolonged Hospital Length of Stay in Adults with Imported Falciparum Malaria—An Observational Study from a Tertiary Care University Hospital in Berlin, Germany
Source: Microorganisms. 2021 Sep 12;9(9):1941. doi: 10.3390/microorganisms9091941 (PMC8466442; doi:10.3390/microorganisms9091941)
Supplement: Supplementary file 1 [file microorganisms-09-01941-s001.zip › microorganisms-1252739-supplementary.pdf]

Factors associated with prolonged hospital length of stay in adults with imported falciparum malaria – an observational study from a tertiary care university hospital in Berlin, Germany - Table S1. STROBE Statement checklist

	Item No.	Recommendation	Page No.	Relevant text from manuscript
**Title and abstract**	1	(*a*) Indicate the study's design with a commonly used term in the title or the abstract	1	Title: "observational study" Abstract: "This retrospective observational study"
		(*b*) Provide in the abstract an informative and balanced summary of what was done and what was found	1	
**Introduction**				
Background/rationale	2	Explain the scientific background and rationale for the investigation being reported	1-2	Data on factors associated with hospital length of stay (LOS) in imported falciparum malaria is scarce and resource allocation has become more importantn as a result of the COVID-19 pandemic.
Objectives	3	State specific objectives, including any prespecified hypotheses	2	Determine the under-explored factors associated with hospital length of stay (LOS) in imported falciparum malaria in order to (1) allow better resource allocation and (2) to identify potential targets for improving management.
**Methods**				
Study design	4	Present key elements of study design early in the paper	2	Retrospective analysis of all cases eligible during the

				study period. Secondary analysis of a previous observational study.
Setting	5	Describe the setting, locations, and relevant dates, including periods of recruitment, exposure, follow-up, and data collection	2	Location: Charité University Hospital, Berlin, a tertiary-care teaching hospital. Study period: January, 1st 2001 through December, 31st 2015.
Participants	6	(*a*) *Cohort study*—Give the eligibility criteria, and the sources and methods of selection of participants. Describe methods of follow-up<br>*Case-control study*—Give the eligibility criteria, and the sources and methods of case ascertainment and control selection. Give the rationale for the choice of cases and controls<br>*Cross-sectional study*—Give the eligibility criteria, and the sources and methods of selection of participants	2	All eligible cases ≥18 years of age hospitalized with slide proven imported falciparum malaria were enrolled (Flow chart: Figure 1). No follow-up was required according to the study design.
		(*b*) *Cohort study*—For matched studies, give matching criteria and number of exposed and unexposed<br>*Case-control study*—For matched studies, give matching criteria and the number of controls per case		
Variables	7	Clearly define all outcomes, exposures, predictors, potential confounders, and effect modifiers. Give diagnostic criteria, if applicable	3	a) Outcome of interest: hospital length of stay, calculated in days by subtracting the day of admission from the day of discharge. Strict WHO 2014 definition for severe falciparum malaria was applied.
Data sources/ measurement	8*	For each variable of interest, give sources of data and details of methods of assessment (measurement). Describe comparability of assessment methods if there is more than one group	2	For all patients standardized electronic files with detailed information on

				sociodemographics, travel history, full medical history including prior malaria episodes, current medication, results of physical examination and laboratory investigations were available. Data capture was therefore high.
Bias	9	Describe any efforts to address potential sources of bias		
Study size	10	Explain how the study size was arrived at	2	All eligible cases ≥18 years of age hospitalized with slide proven imported falciparum malaria during the study period were enrolled. Exclusion criteria were repeat episodes and incomplete records.

Quantitative variables	11	Explain how quantitative variables were handled in the analyses. If applicable, describe which groupings were chosen and why	4	Only non-parametric tests were used. Categorical data were compared by chi^2 test, while the Mann-Whitney-U-test was used for continuous data. Conventional Cox proportional hazard regression with censoring of cases discharged after the median of LOS (i.e., by day 3) was used in univariate and multivariate analysis. Covariates violating the

				proportional hazard assumption were excluded from multivariate analysis. For variable selection the best subset selection method was used. The results from the final multivariate model were reported as adjusted hazard ratios (aHRs). The proportional hazard assumption was again tested for each covariate in the final multivariate model and for the global model using the Schoenfeld residual test. Influential observations were tested by dfbeta values. The statistical significance level was set at 5% for all analyses.
Statistical methods	12	(*a*) Describe all statistical methods, including those used to control for confounding	4	See above (item #11).
		(*b*) Describe any methods used to examine subgroups and interactions		Not applicable.
		(*c*) Explain how missing data were addressed		There were no missing data for the outcome of interest.
		(*d*) *Cohort study*—If applicable, explain how loss to follow-up was addressed<br>*Case-control study*—If applicable, explain how matching of cases and controls was addressed<br>*Cross-sectional study*—If applicable, describe analytical methods taking account of sampling strategy		Not applicable.
		(*e*) Describe any sensitivity analyses	4	To ensure the robustness of the investigation sensitivity analyses using other thresholds (i.e., discharge after 4 and 7

				days) were performed. Only covariates with significant associations with LOS consistent in all three analyses were included in the following multivariate analysis.
**Results**				
Participants	13*	(a) Report numbers of individuals at each stage of study—eg numbers potentially eligible, examined for eligibility, confirmed eligible, included in the study, completing follow-up, and analysed	5	Reported under section 3.1. and summarized in Figure 1.
		(b) Give reasons for non-participation at each stage	2, 4	Exclusion criteria were repeat episodes (n = 22) and incomplete records (n =1) data.
		(c) Consider use of a flow diagram	2, 4	A flow diagram is presented as Figure 1.
Descriptive data	14*	(a) Give characteristics of study participants (eg demographic, clinical, social) and information on exposures and potential confounders	6-8	Baseline characteristics are summarized in Table 2.
		(b) Indicate number of participants with missing data for each variable of interest	6-8 and 8-10	For each covariate the available case numbers are given in Table 2 and 3.
		(c) *Cohort study*—Summarise follow-up time (eg, average and total amount)		Not applicable.
Outcome data	15*	*Cohort study*—Report numbers of outcome events or summary measures over time	7, 8-10	Reported under section 3.2. and Table 3.
		Case-control study—Report numbers in each exposure category, or summary measures of exposure		Not applicable.
		Cross-sectional study—Report numbers of outcome events or summary measures		Not applicable.
Main results	16	(*a*) Give unadjusted estimates and, if applicable, confounder-adjusted estimates and their precision (eg, 95% confidence interval). Make clear which confounders were adjusted for and why they were included	7, 8-10, 10	All unadjusted estimates are summarized in Table 3, with 95% confidence intervals given for each covariate. The main unadjusted estimates are

				outlined in section 3.2. and illustrated in Figure 3. One covariate was excluded from multivariate analysis in order to avoid overfitting, another one had to be excluded because the proportional hazard assumption was violated.
		(*b*) Report category boundaries when continuous variables were categorized		Not applicable.
		(*c*) If relevant, consider translating estimates of relative risk into absolute risk for a meaningful time period		Not applicable.
Other analyses	17	Report other analyses done—eg analyses of subgroups and interactions, and sensitivity analyses	8-10	Sensitivity analyses were an important part of the statistical investigation and results are summarized in Table 3.
**Discussion**				
Key results	18	Summarise key results with reference to study objectives	11-13	Key results of the analysis are: (1) with virtually unlimited resources and high standard of care mortality from imported falciparum malaria can be kept to a minimum. (2) To achieve this task prolonged hospitalization is required for some patients, particularly those with severe malaria and multi-organ involvement. (3) The factors associated with prolonged LOS are not the complications directly related to

				sequestration of infected and uninfected erythrocytes in the microcirculation (metabolic acidosis and coma), but the complications typically occurring later or lasting longer in the course of the disease, namely shock, respiratory distress and acute kidney injury stage 3 requiring renal replacement therapy.
Limitations	19	Discuss limitations of the study, taking into account sources of potential bias or imprecision. Discuss both direction and magnitude of any potential bias	13	The main limitations of the study are the retrospective, single-centre design and the long observation period. This limits the quality of the data and thus generalizability. Yet, the study is intended to analyse an "ideal-world scenario" for the treatment of falciparum malaria in order to identify the factors that require most resources under the best possible conditions. Generalizability is therefore per se limited.
Interpretation	20	Give a cautious overall interpretation of results considering objectives, limitations, multiplicity of analyses, results from similar studies, and other relevant evidence	11-13	Done in the "Discussion" section.
Generalisability	21	Discuss the generalisability (external validity) of the study results	13	Generalisability is discussed in the "limitations" section.
**Other information**				
Funding	22	Give the source of funding and the role of the funders for the present study and, if applicable,	14	This work received no funding.

		for the original study on which the present article is based

*Give information separately for cases and controls in case-control studies and, if applicable, for exposed and unexposed groups in cohort and cross-sectional studies.

Note: An Explanation and Elaboration article discusses each checklist item and gives methodological background and published examples of transparent reporting. The STROBE checklist is best used in conjunction with this article (freely available on the Web sites of PLoS Medicine at http://www.plosmedicine.org/, Annals of Internal Medicine at http://www.annals.org/, and Epidemiology at http://www.epidem.com/). Information on the STROBE Initiative is available at www.strobe-statement.org.
